# Research on the influence of personalized principles in AR educational resources on the learning effectiveness of college students

**DOI:** 10.3389/fpsyg.2025.1618990

**Published:** 2026-01-14

**Authors:** Jingsheng Zeng, Dayu Fan, Hanlin Hu, Guan Huang, Haohua Zhang

**Affiliations:** 1 Wu Tong Qiao Middle School, Leshan, Sichuan, China; 2 Maerkang High School of Sichuan Province, Maerkang, Sichuan, China; 3 School of Education, China West Normal University, Nanchong, Sichuan, China

**Keywords:** AR educational resources, personalized principle, learning effect, AR, learning space, personalized narrative style

## Abstract

With the rapid development of modern educational technology, the application of augmented reality (AR) technology in education is gradually showing its potential. While building innovative teaching environments and experiential learning scenarios, abstract concepts are visualized to create a learning space that combines interactivity and immersion. Through gamified design elements and incentive mechanisms, learners' participation and learning effectiveness are effectively enhanced, bringing new educational possibilities and research dimensions. This study is supported by theories such as multimedia learning cognitive emotion theory, embodied cognition theory, and cognitive load theory, and independently developed two AR educational resources. Based on the theme nature of learning materials (neutral/negative) and learner characteristics (high/low spatial ability), a two-dimensional division is carried out, and a quasi experimental research method is adopted. Eye tracking technology and brainwave technology are comprehensively used to collect physiological data of learners from multiple dimensions such as visual attention allocation, cognitive load, and emotion. The learning process and cognitive mechanism boundary conditions of learners in different types of AR materials are analyzed in depth. Research has found that personalized storytelling styles have different effects on maintaining and transferring high and low spatial abilities, while formal storytelling styles have different effects on subjective psychological effort, cognitive use, learning interest, positive emotions, and attention toward high and low spatial abilities.

## 1 Introduction

Educational informatization as a crucial force driving the reform of China's education system, has entered a crucial stage of transformation and upgrading. The Chinese government has continuously issued important documents such as the “〈〈Education Informatization 2.0 Action Plan〉〉” (Ministry of Education), “China Education Modernization 2035” (The Central Committee of the Communist Party of China) and the “14th Five-Year Plan Outline” (The Central Cybersecurity and Information Technology Commission) further implementing the “Internet+ Education” plan and deploying ten major strategic tasks for education modernization. These documents propose advancing the “Digital China” goal. As educational resources constantly evolve and update, augmented reality (AR) technology empowers educational informatization by significantly improving users' perception of space and time. By visualizing the real and virtual worlds simultaneously, AR technology helps learners gain a deeper understanding of abstract concepts and their interrelationships. “AR+ Education” has emerged as a research hotspot in the education field. AR technology is relatively easy to apply, has a wide range of applications, and high sustainability. It is suitable for all educational stages from preschool to university and is an important cutting-edge direction for the next generation of information technology. Therefore, how to effectively integrate AR technology with education, design and develop AR educational resources that meet current learning needs, and construct AR learning environments suitable for learners have become important research topics.

## 2 Research status of augmented reality technology in the field of education

### 2.1 Research status in China

**Figure 1:**
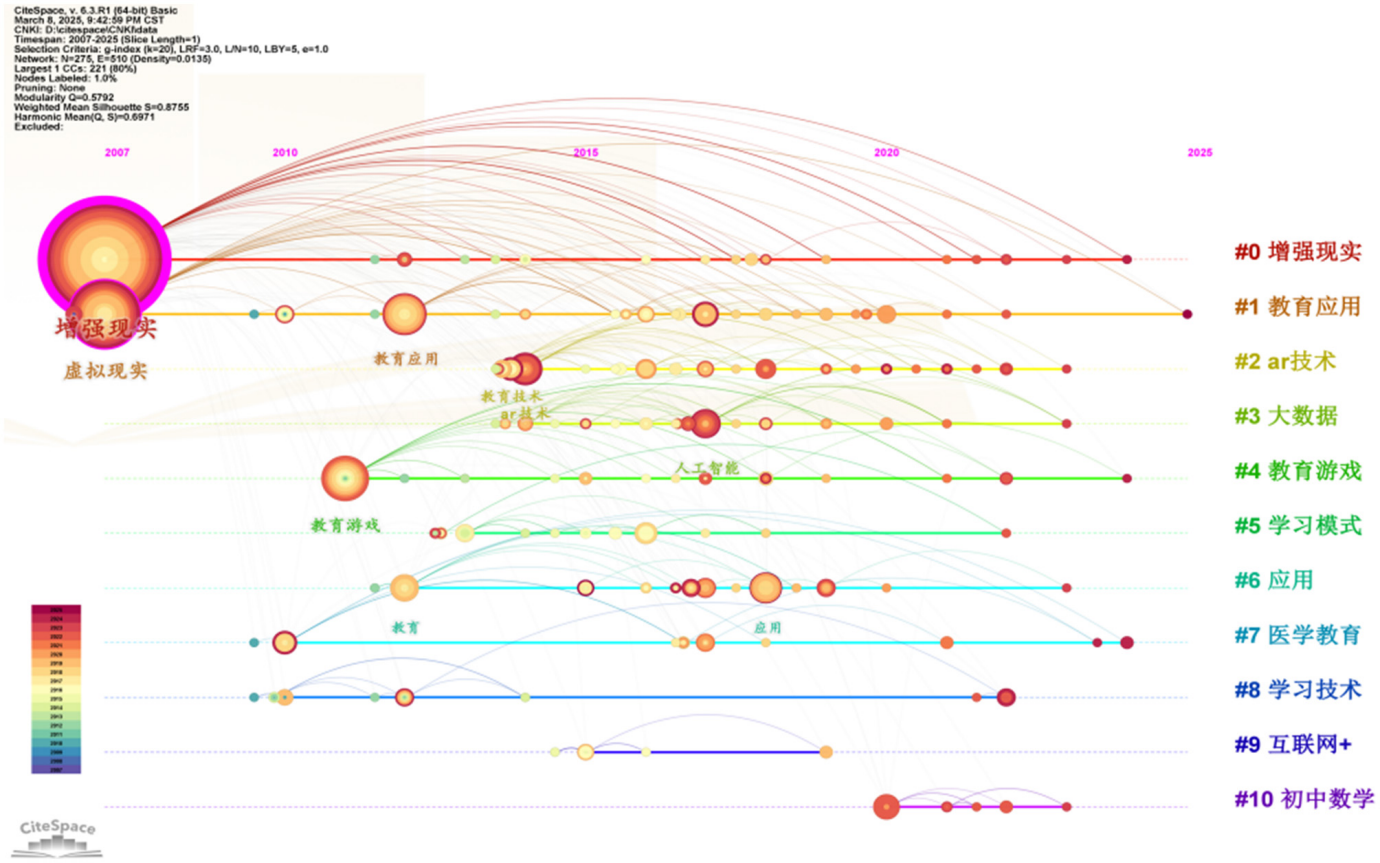
Timeline of augmented reality + education keyword clustering.

By searching for “augmented reality + education” as the theme in the China National Knowledge Infrastructure (CNKI) database and importing the literature into the CiteSpace software, the time-line map of keyword clustering is shown in Figure 1.

The research focused on the educational application and development of AR educational resources. Through sorting and analyzing the literature, representative research results were organized into Tables 1, 2.

**Table 1:** Literature review of research on discipline teaching and children's education applications.

**Personnel**	**Research process and purpose**	**Research methodology**	**Evaluation indicators**
Qian et al. (2024)	Explore the impact of the Understanding-Based Learning Approach (UBAR) on students' writing performance and engagement, as well as the learning satisfaction of teachers and students	Quasi-experimental research method	Academic performance, learning engagement, learning satisfaction
Feng et al. (2023)	Compare the learning effects of students using AR, video, and PPT for learning geography knowledge, and measure physiological and psychological data	Quasi-experimental research method	Learning outcomes, physiological acceptability, cognitive load
Zhu et al. (2023)	Apply AR technology to senior high school chemistry education and find that AR technology has a significant immediate teaching effect and good teaching retention effect on students' learning of microscopic structure knowledge	Quasi-experimental research method	Immediate teaching effect, teaching retention effect
Yuan et al. (2019)	Apply AR technology to geography teaching and compare the teaching effects of AR 3D video and traditional 2D video	Quasi-experimental research method	Learning outcomes
Zhang and Jiang (2018)	Prospects for the application of AR technology in science education	Questionnaire survey method	Higher-order thinking

**Table 2:** Literature review of research on the design and development of educational resources.

**Personnel**	**Research process and purpose**	**Research methodology**	**Evaluation indicators**
Cai et al. (2023)	Develop an AR science inquiry tool based on a lightweight brain-computer interface and compare the learning outcomes of participating in AR science inquiry activities with and without a brain-computer interface	Quasi-experimental research method	Science inquiry performance, self-efficacy of participation
Wang and Li (2020)	Develop a teaching resource for children's Chinese learning based on AR technology	Quasi-experimental research method	Academic performance
Wang and Liu (2019)	Propose new concepts and methods for optimizing AR learning resources from the perspective of multimedia language	Quasi-experimental research method	learning experience
Tian et al. (2019)	Design and develop a mobile AR game for preschool children	Quasi-experimental research method	learning experience
Ye et al. (2018)	Design an AR sports game and compare its teaching effects with traditional teaching methods	Quasi-experimental research method	Teaching effects
Chen and Wan (2017)	Develop an AR English vocabulary learning game and apply it	Quasi-experimental research method	learning experience

Currently, research on AR education in China is in its infancy, mainly focusing on classroom applications in senior high school subjects such as Chinese, geography, chemistry, and science. Evaluation indicators mainly include academic performance, learning engagement, cognitive load, and higher-order thinking skills. Research has not yet formed a systematic and holistic framework, with scattered and single evaluation dimensions.

### 2.2 Research status abroad

Research on AR in education abroad pays more attention to the environment where virtual objects are superimposed on the real world. Hsin-Yi Chang provides superimposed virtual objects in the campus environment to solve practical problems (Chang et al., 2016). AR is used to enhance the learning experience of museum visits in the real world (Meekaew and Watcharee, 2021), Hsien-Sheng Hsiao uses virtual objects to enhance materials for classroom learning (Hsiao et al., 2016). Previous studies have shown that AR teaching has a positive impact on three different levels of learning motivation and attitude, academic performance, and learning performance. Lu and Liu (2015) designed a learning scheme based on digital games combined with AR technology to enhance children's learning ability and learners' motivation. Zeynep et al. (2018) showed that mobile AR technology has improved learners' knowledge and skills in geography education. Bursali and Yilmaz (2019) found that students who participated in reading activities using AR applications exhibited higher levels of reading comprehension and learning persistence compared to those who used traditional methods. Research has shown that AR is effective for learning, but there has been little exploration of how to use AR for learning and when it is effective, with more media comparison studies published since 2009 compared to other types of research (Buchner and Kerres, 2023).

It can be seen that researchers should pay attention to the impact of AR technology on learners of different genders and prior knowledge, and should compare two different AR learning applications of procedural knowledge and declarative knowledge, and can also combine the above two aspects.

### 2.3 Research review

Based on the research on AR technology in the field of education at home and abroad, it can be seen that teaching based on this technology has a positive impact on learners' learning outcomes and has great application value. However, it is not difficult to find that many studies focus on comparing the effect of AR technology on learning with traditional learning methods or other technologies. At present, there is a lack of research on when and how to use AR technology for education and learning, which is the direction for future research. AR technology is mainly used on computers, tablets, and smartphones, which greatly improves its convenience of application. Therefore, this study developed two AR learning resources based on mobile devices and applied them in teaching to explore their impact on learners' learning outcomes.

## 3 Research design

### 3.1 Overall experimental design

Experiment 1 explored the impact of the personalization principle on learning outcomes when learning neutral-themed AR materials. Experiment 2 explored the impact of the personalization principle on learning outcomes when learning negative-themed AR materials. The overall framework of the experiment is shown in Table 3.

**Table 3:** Overall framework of the experiment.

**Experimental project**	**Learning material theme**	**Independent variables**	**Dependent variables**
Experiment 1	Neutral (heart structure and blood circulation)	2 (low spatial ability, high spatial ability) × 2 (formal style, personalized style)	Academic performance, cognitive load, attention
Experiment 2	Negative (types and causes of cerebral hemorrhage)	2 (low spatial ability, high spatial ability) × 2 (formal style, personalized style)	Academic performance, cognitive load, attention, emotions

### 3.2 Experimental subjects

The experiment recruited 166 subjects from a normal university. All subjects had normal hearing and vision or corrected vision, and more than 90% of the subjects had no experience using AR for learning. All subjects underwent spatial ability tests and formal experiments. Abnormal EEG and eye-tracking data were excluded, and 120 subjects were selected as valid subjects for this experiment, including 60 with high spatial ability and 60 with low spatial ability. The age distribution was between 19 and 23 years old, with 34 males and 86 females. After introducing the experiment content and process to the subjects and obtaining their consent, they were randomly divided into 8 groups. At the end of the experiment, they were given a gift. The gift includes commemorative medals, plush toys, keychains, and snacks.

### 3.3 Measurement methods and tools

The measurement of prior knowledge used a 7-point Likert scale, see Appendix 1. The learner's spatial ability measurement scale refers to the spatial ability test scale developed by Luo (2014), see Appendix 2. The measurement of learning performance in the experiment included retention performance and transfer performance, see the specific questions in Appendix 3. The subjective measurement tool for cognitive load used the self-evaluation scale developed by Paas and Van Merriënboer (1994), see Appendix 4; objective cognitive load was reflected by the brain wave instrument recording the brain engagement, with the lowest value being “−100” and the highest value being “100”. The subjective emotional measurement used the Positive and Negative Affect Scale (PANAS) developed by Watson et al. (1988), see Appendix 5. Objective emotional measurement was reflected by the relaxation degree recorded by the brain wave instrument, with the lowest value being “0” and the highest value being “100”.

### 3.4 Experimental data recording and export

**Figure 2:**
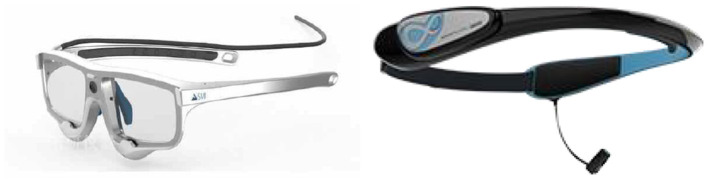
Eye movement and brain wave devices used in the study.

**Figure 3:**
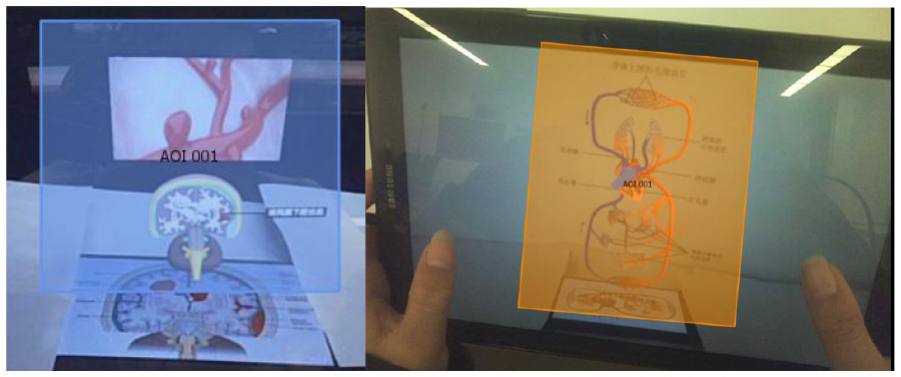
Division of interest areas.

This study used an eye-tracking instrument and a brain wave instrument, as shown in Figure 2. The BeGaze 3.7 software of the SMI eye-tracking instrument divided the areas of interest (as shown in Figure 3), and the iViewETG software collected and exported eye-tracking data. The BrainLink Lite Pro portable brain wave instrument recorded attention, relaxation, and brain engagement while the subjects were watching the video, and the data was collected and exported by the companion APP.

### 3.5 Experimental process

**Figure 4:**
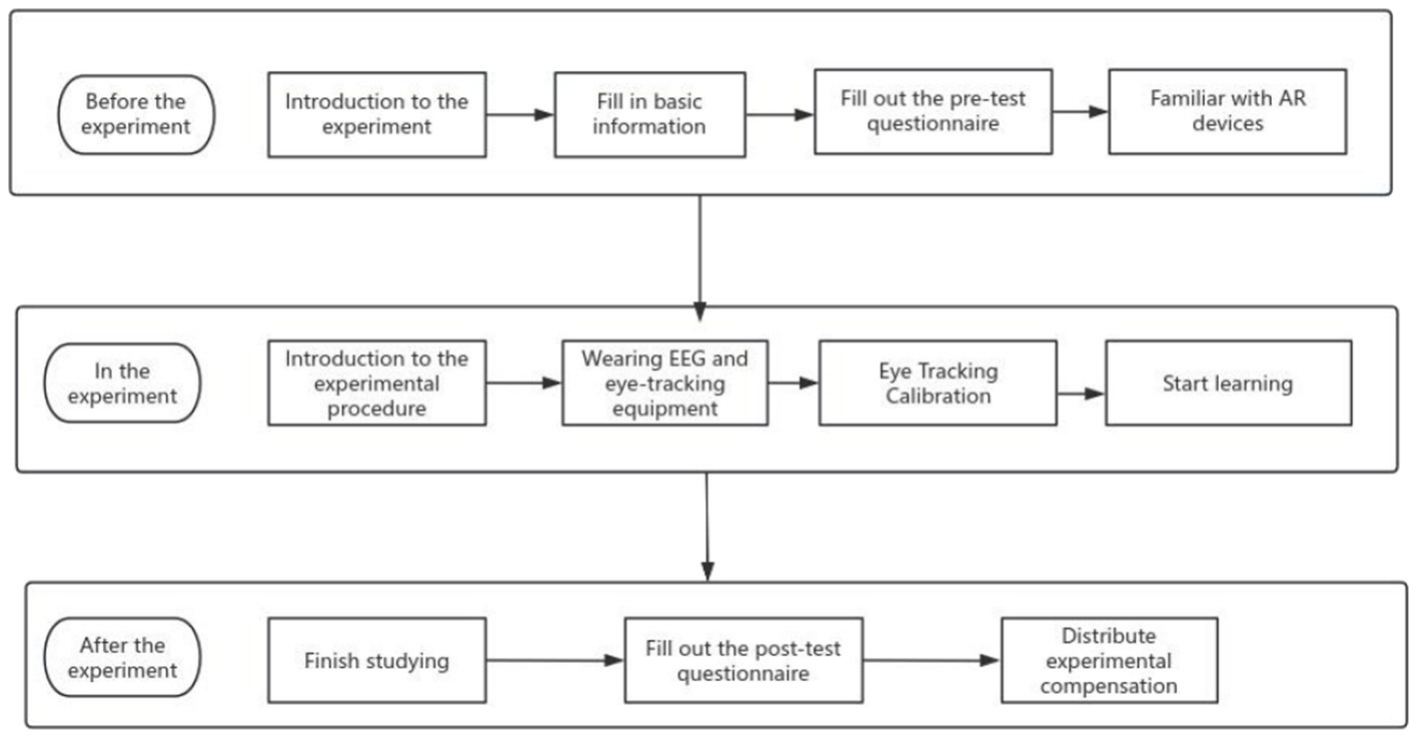
Experimental flowchart.

**Figure 5:**
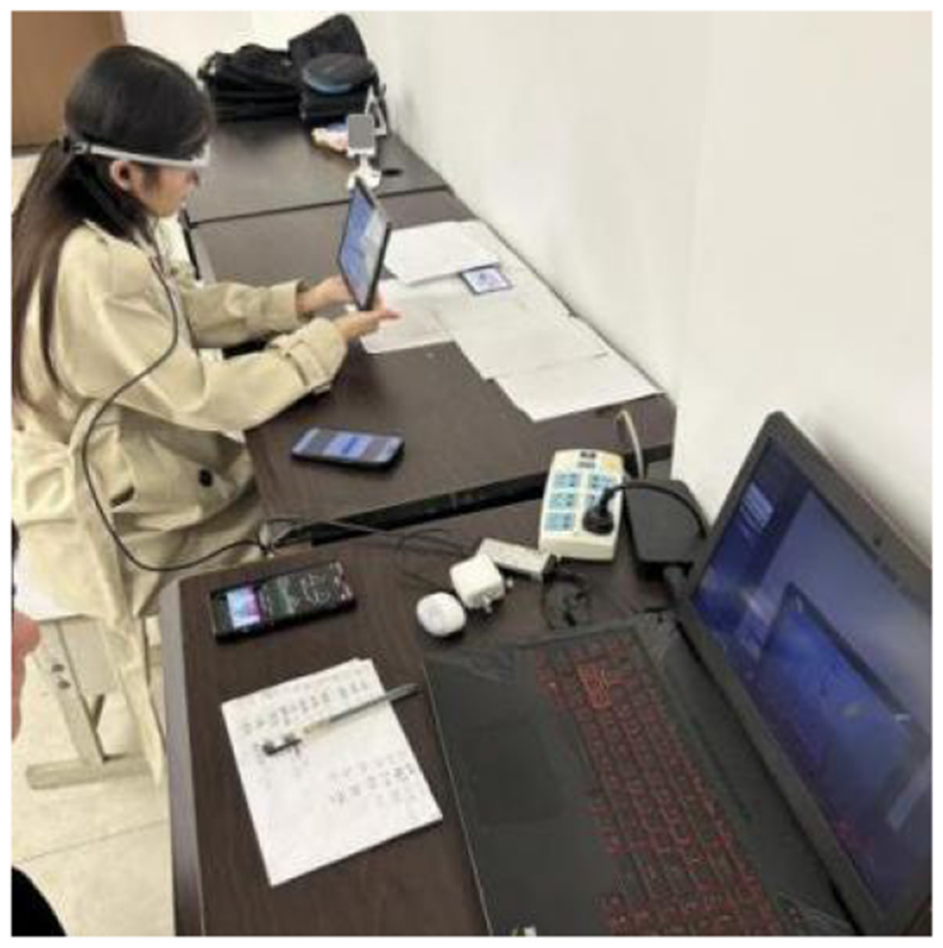
Experimental site map.

The experimental part of this study included three stages. The experiment process is shown in Figure 4, and the experiment site is shown in Figure 5.

Experiment preparation stage:

(1) Design, develop and debug AR learning resources.
(2) Design and modify the questionnaire, and debug the brain wave instrument and eye movement instrument.
(3) Design the formal experimental process, determine the experimental site, waiting place, time, questionnaire filling place, etc.
(4) Call the students in the same group to conduct the pre experiment and simulate the steps of the formal experiment to ensure that they are correct.

Experiment implementation phase:

(1) Recruit experimental subjects from the whole school, establish a QQ group, issue online forms, fill in the time and number.
(2) The subjects filled in the pretest questionnaire and informed them of the experimental process and precautions.
(3) The experimenters wore and calibrated the brain wave meter and eye movement meter for the subjects, and introduced how to operate the learning resources.
(4) The subjects began to study independently and filled out the posttest questionnaire.

Analysis and discussion of experimental results:

The experimental data were exported from the software, sorted out, and imported into SPSS 26 for descriptive statistics and analysis of variance. Finally, the conclusion was drawn for discussion.

### 3.6 Experimental materials

Learners scan the recognition diagram, wear headphones to listen to audio explanations, and cooperate with the models that appear on the tablet computer screen for learning. The audio explanation of the personalized narrative style group adds personal pronouns based on the formal style. The two groups of experimental materials only differ in audio explanation, and the learning content and pictures are the same.

Neutral materials refer to learning materials that do not have obvious emotional tendencies or colors, and usually do not trigger strong emotional reactions. When learners come into contact with these materials, they do not experience significant positive or negative emotions. This type of learning material is mainly used to convey knowledge, information, or skills, and does not involve emotional guidance or regulation. For example, the formation principle of lightning, knowledge related to botany, computer technology, etc., all fall within the scope of neutral materials.

Negative materials refer to learning materials that have negative emotional tendencies or may trigger negative emotions, usually containing content that is unsettling, sad, anxious, or angry. When learners come into contact with these materials, they may experience negative emotions such as sadness, anxiety, fear, or anger. For example, knowledge about cerebral hemorrhage, hepatitis, breast cancer, etc.

In this study, neutral materials describe the specific structure of the heart and the pathways of blood circulation, while negative materials describe the location and corresponding symptoms of cerebral hemorrhage.

(1) Neutral Experimental Materials

**Figure 6:**
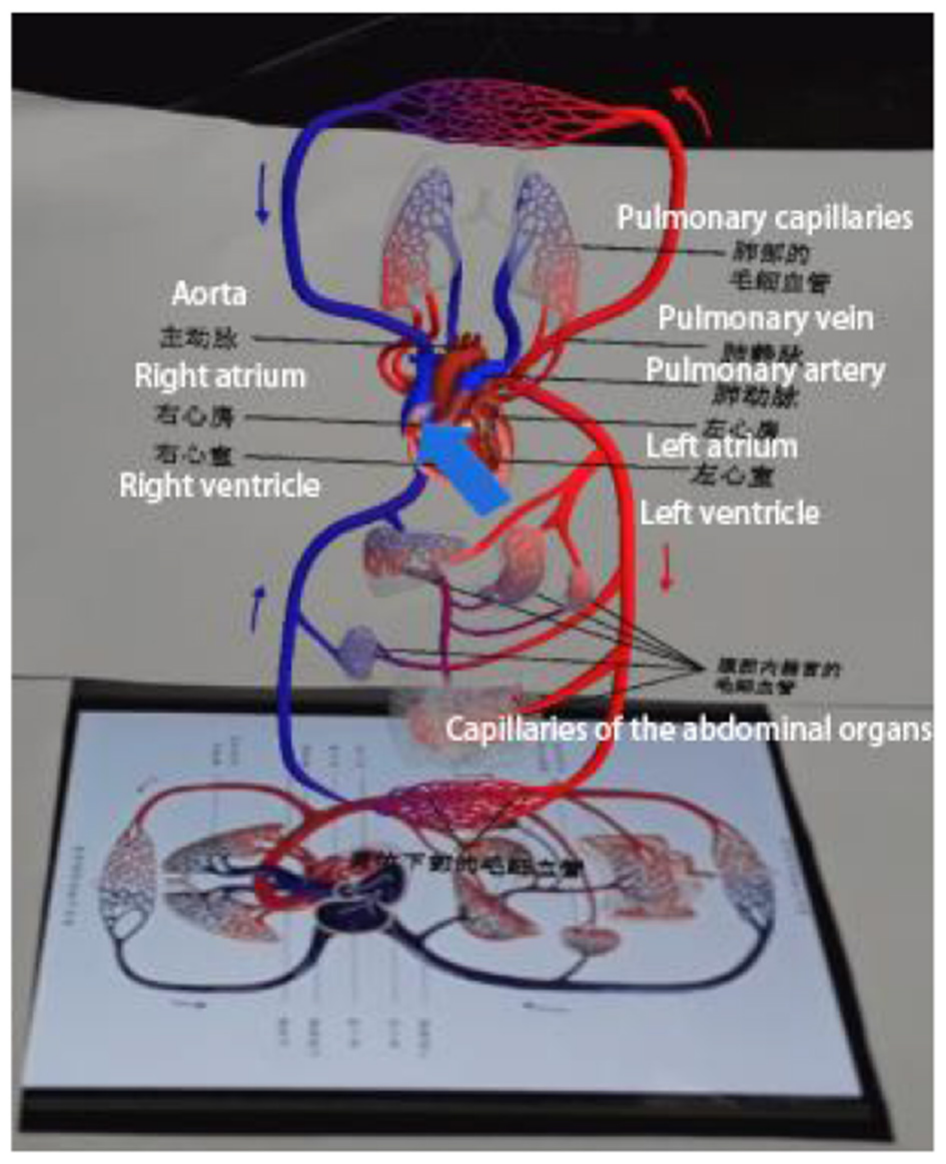
Arrow indication screen.

**Figure 7:**
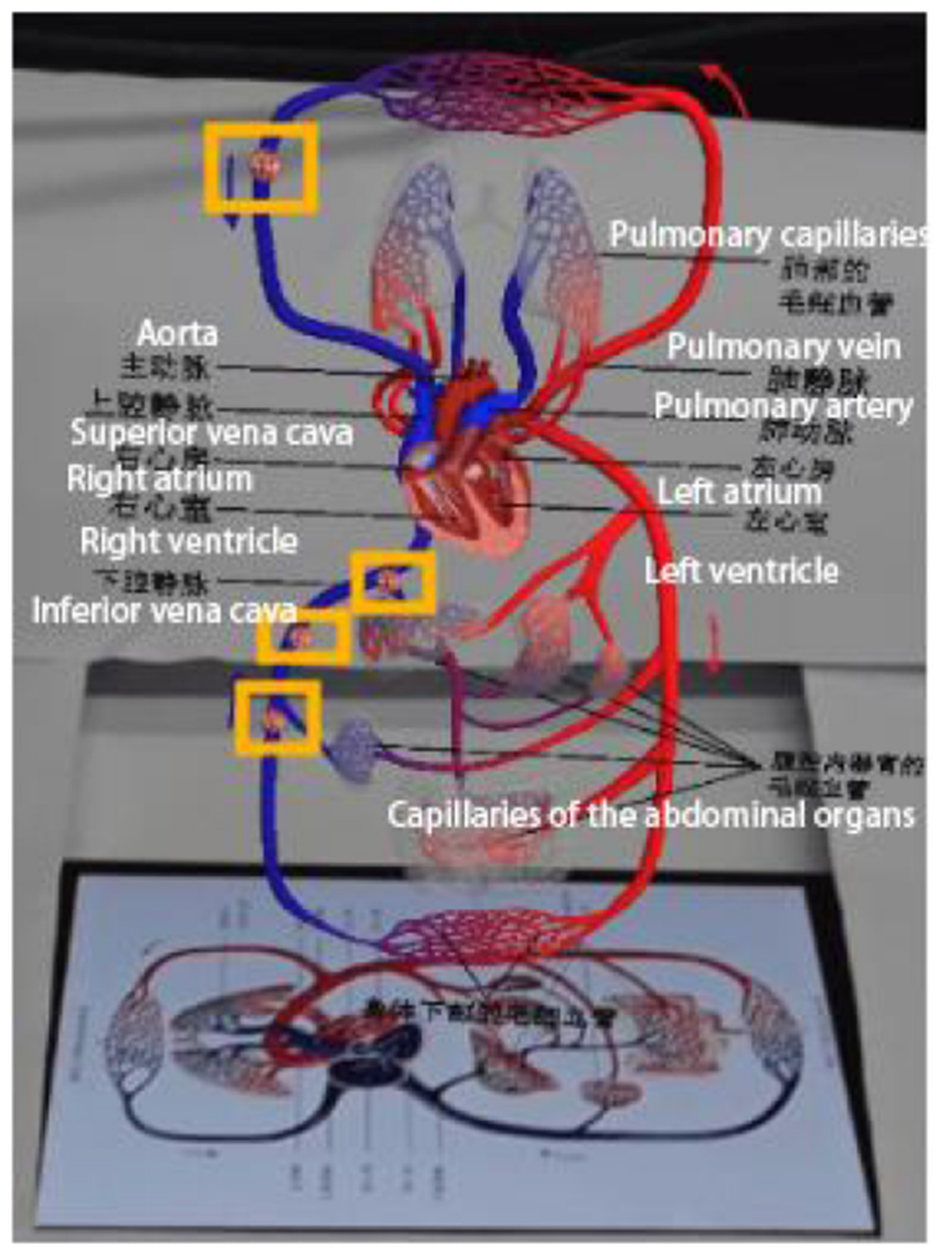
Simulated blood flow screen.

Experiment 1 mainly introduces the specific structure of the human heart and the path of blood circulation. Arrows will appear during the process to guide learners' attention, as shown in Figure 6; animations will also appear to simulate the direction of blood flow, as shown in Figure 7.

The learning duration of formal style group and personalized narrative style group were 3 min 24 s and 4 min 10 s respectively

(2) Negative Experimental Materials

**Figure 8:**
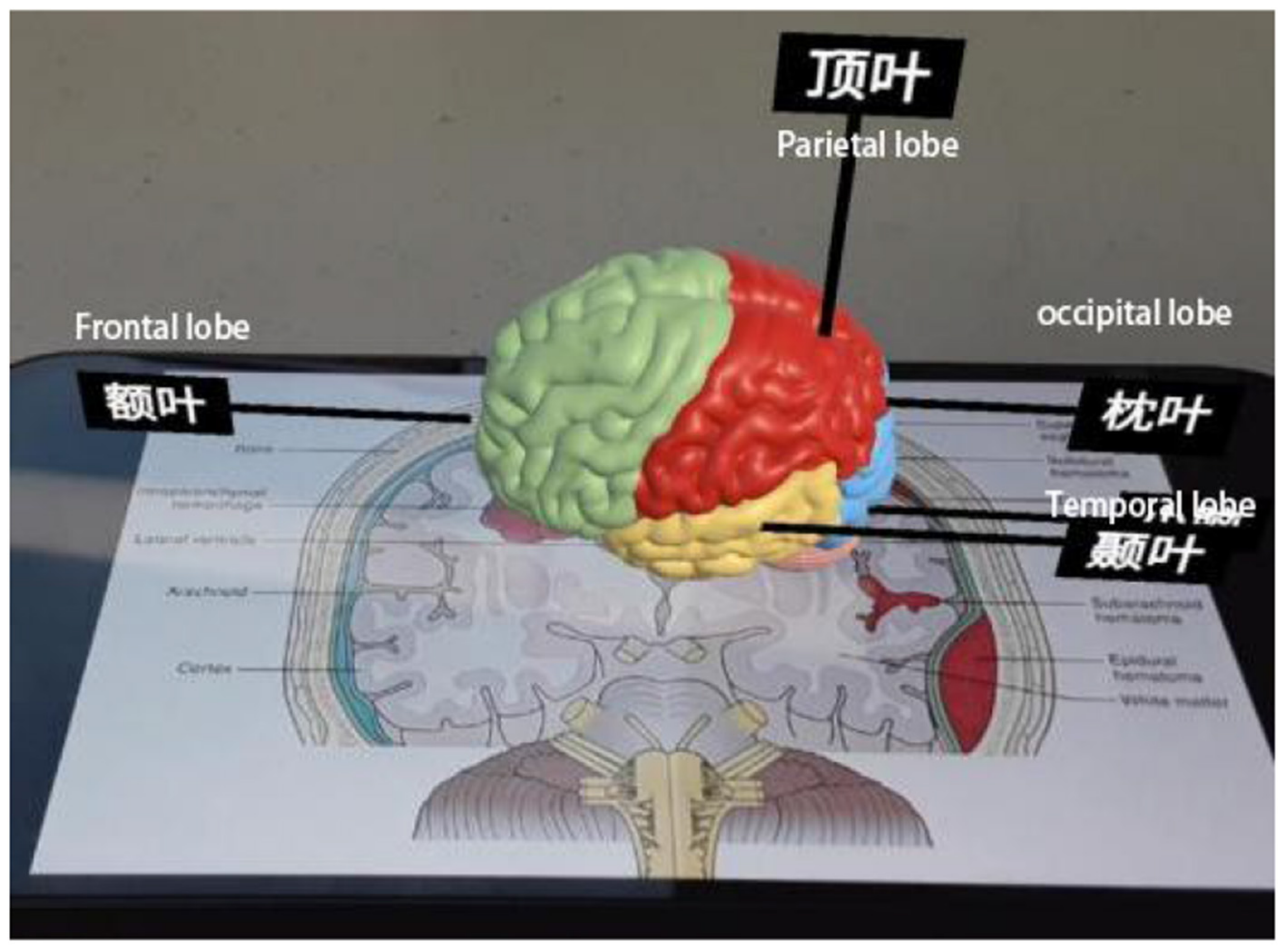
Explanation of cerebral cortex knowledge.

**Figure 9:**
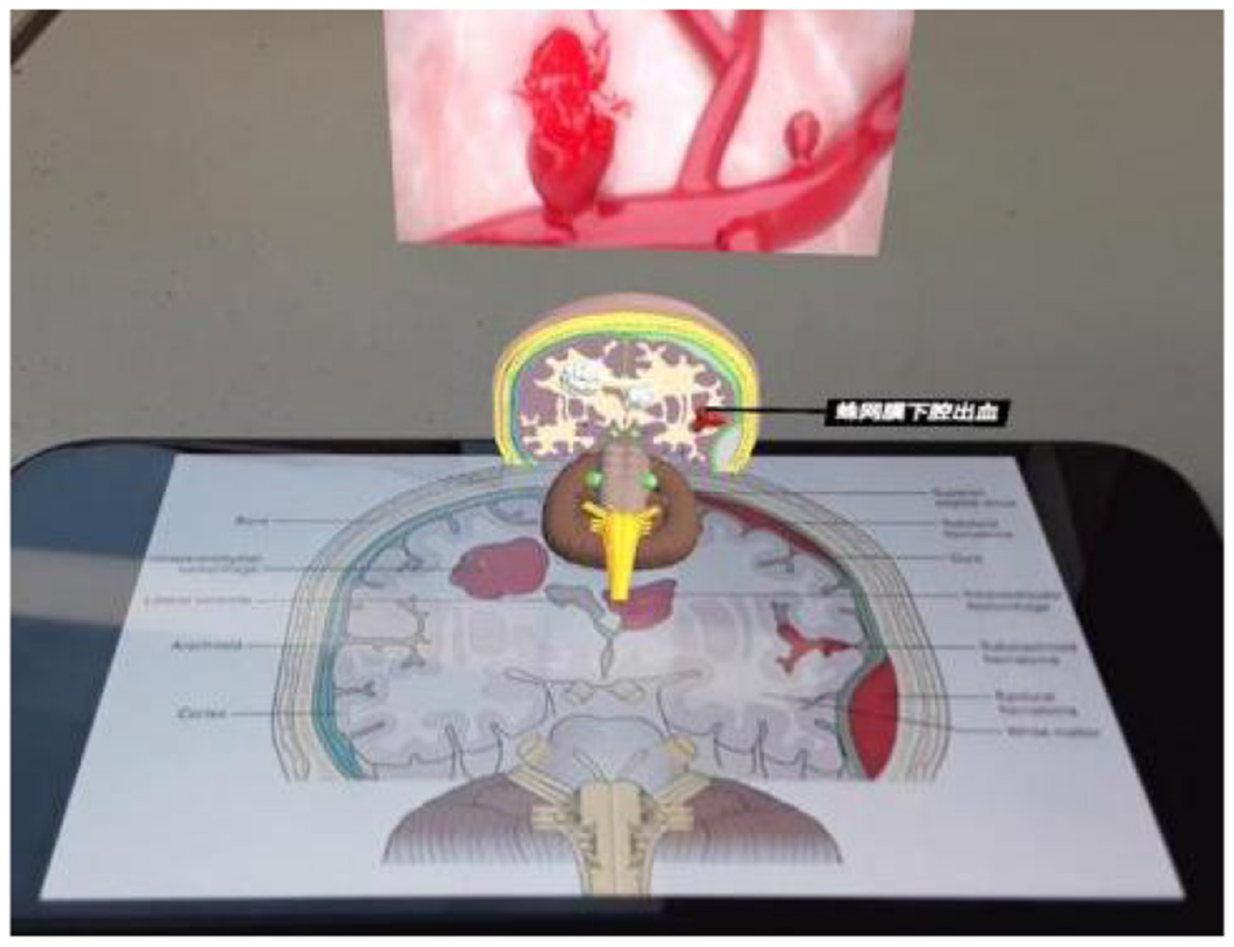
Explanation of cerebral hemorrhage related knowledge.

Experiment 2 mainly introduces the basic structure of the human brain and the symptoms and causes of cerebral hemorrhage. During the learning process, color changes and text explanations will appear to guide learners' attention, as shown in Figure 8; AR videos will also appear to help learners understand and remember, as shown in Figure 9.

The learning duration of formal style group and personalized narrative style group were 3 min 47 s and 3 min 51 s respectively.

## 4 Experimental process and result analysis

### 4.1 Experiment 1: exploring the impact of the personalization principle on learning outcomes in neutral materials

This experiment mainly explores the impact of the personalization principle on the learning outcomes of learners with different spatial abilities when learning neutral learning materials. The independent variables of the experiment were: 2 (high, low spatial ability) × 2 (personalized style, formal style), and the dependent variables were: learning performance, cognitive load, and attention.

(1) Experimental Hypotheses

The influence of narrative style on learners' academic performance, cognitive load and attention in augmented reality environment:

H1a Under the influence of different narrative style, learners' performance is significantly different, and personalized narrative style is significantly higher than formal style.
H1b Under the influence of different narrative styles, learners' transfer performance is significantly different, and personalized narrative style is significantly higher than formal style.
H2 Learners' cognitive load is significantly different under the influence of different narrative styles, and personalized narrative style is significantly lower than formal style.
H3 Learners' attention is significantly different under the influence of different narrative styles, and the personalized narrative style is significantly higher than the formal style.

The effects of learners' spatial ability on academic performance, cognitive load and attention in augmented reality environment:

H4a Learners with different spatial abilities have significant differences in retention performance, and learners with high spatial abilities are significantly higher than those with low spatial abilities.
H4b Learners with different spatial abilities have significant differences in transfer performance, and learners with high spatial abilities are significantly higher than those with low spatial abilities.
H5 Learners with different spatial abilities have significant differences in cognitive load, and learners with high spatial abilities are significantly lower than those with low spatial abilities.
H6 Learners with different spatial abilities have significant differences in attention, and learners with high spatial abilities are significantly higher than those with low spatial abilities.

(2) Analysis of academic performance

This study used the subjects' retention and transfer scores as dependent variables, learners' spatial ability and learning material presentation style as independent variables, and previous test scores as covariates. SPSS26 was used for two factor analysis of variance, and the descriptive statistics of each data item are shown in Table 4.

**Table 4:** Descriptive statistical results.

**Score type**	**spatial ability**	**narrative style**	**N**	**M**	**SD**
Prior knowledge	High spatial ability	Personalized style	15	7.13	1.355
		Formal Style	15	7.33	1.543
	Low space capability	Personalized style	15	7.13	1.407
		Formal Style	15	7.86	1.807
Maintain grades	High spatial ability	Personalized style	15	10.27	1.831
		Formal Style	15	9.00	2.330
	Low space capability	Personalized style	15	9.60	3.250
		Formal Style	15	7.33	2.380
Transfer score	High spatial ability	Personalized style	15	2.47	0.743
		Formal Style	15	1.47	1.125
	LOW space capability	Personalized style	15	2.13	0.990
		Formal Style	15	1.53	1.505

Comparing the scores of the prior knowledge of the four groups of learners as the dependent variable, the results of one-way ANOVA showed that there was no significant difference in the prior knowledge of the four groups of learners (*F* = 0.761, *P* = 0.521 > 0.05), as shown in Table 5. Therefore, there is no significant difference in the level of prior knowledge among the four groups of learners, and the experiment can be carried out.

**Table 5:** Results of variance analysis of learners' prior knowledge.

**Dependent variable**	**Sum of squares**	**Mean square**	***F***	**Significance**
Prior knowledge	5.400	1.800	0.761	0.521

The two factor variance analysis of learning retention performance and transfer performance showed that the main effect of spatial ability was not significant in retention performance (*F* = 3.268, *P* = 0.076 > 0.05), the main effect of narrative style was significant (*F* = 7.494, *P* = 0.008 < 0.05), and the interaction effect of spatial ability and narrative style was not significant (*F* = 0.6, *P* = 0.442 > 0.05); In terms of transfer performance, the main effect of spatial ability was not significant (*F* = 0.211, *P* = 0.648 > 0.05), the main effect of narrative style was significant (*F* = 7.579, *P* = 0.008 < 0.05), and the interaction effect of spatial ability and narrative style was not significant (*F* = 0.474, *P* = 0.494 > 0.05), as shown in Table 6.

**Table 6:** Results of variance analysis of learners' posttest scores.

**Independent variable**	**Dependent variable**	**Sum of squares**	**Mean square**	***F***	**Significance**
Space capability	Maintain performance	20.417	20.417	3.268	0.076
	Migration achievements	0.267	0.267	0.211	0.648
Narrative style	Maintain performance	46.817	46.817	7.494	0.008
	Migration achievements	9.600	9.600	7.579	0.008
Space capability ^*^ narrative style	Maintain performance	3.750	3.750	0.600	0.442
	Migration achievements	0.600	0.600	0.474	0.494

Further paired analysis and comparison of intersubjective effects found that, in terms of maintaining performance, the main effect of narrative style in the personalized style group was significantly higher than that in the formal style group (*P* = 0.008 < 0.05), and the interaction effect of narrative style and spatial ability in the personalized style group was significantly higher than that in the formal style group (*P* = 0.016 < 0.05) when learners were low in spatial ability. In terms of transfer performance, in the main effect of narrative style, the personalized style group was significantly higher than the formal style group (*P* = 0.008 < 0.05). In the interaction effect of narrative style and spatial ability, when learners were high spatial ability, the personalized style group was significantly higher than the formal style group (*P* = 0.018 < 0.05).

(3) Cognitive load analysis

In this study, the subjective questionnaire of cognitive load filled in by the subjects and the degree of brain use measured by the brain wave instrument were used as dependent variables, and the learners' spatial ability and learning material presentation style were used as independent variables. Spss26 was used for two-way ANOVA. The descriptive statistics of the data are shown in Table 7.

**Table 7:** Descriptive statistical results of cognitive load.

**Dependent variable**	**Space capability**	**Narrative style**	***N***	***M***	**SD**
Subjective cognitive load	High space capability	Personalized style	15	9.67	1.799
		Formal Style	15	11.47	3.563
	Low space capability	Personalized style	15	11.60	2.354
		Formal style	15	10.40	2.995
Use brainpower	High space capability	Personalized style	15	44.93	16.283
		Formal style	15	51.08	16.891
	Low space capability	Personalized style	15	38.19	11.845
		Formal style	15	53.88	16.233

The cognitive load includes subjective cognitive load and brain use. Two factor analysis of variance found that in terms of subjective cognitive load, the main effect of spatial ability was not significant (*f* = 0.370, *p* = 0.545 > 0.05), the main effect of narrative style was not significant (*f* = 0.177, *p* = 0.675 > 0.05), and the interaction effect of spatial ability and narrative style was significant (*f* = 4.434, *p* = 0.04 < 0.05); In terms of brain use, the main effect of spatial ability is not significant (*f* = 0.243, *p* = 0.624 > 0.05), the main effect of narrative style is significant (*f* = 7.502, *p* = 0.008 < 0.05), and the interaction effect of spatial ability and narrative style is not significant (*f* = 1.427, *p* = 0.237 > 0.05), as shown in Table 8.

**Table 8:** Results of variance analysis of cognitive load.

**Independent variable**	**Dependent variable**	**Sum of squares**	**Mean square**	***F***	**Significance**
Space capability	Subjective cognitive load	2.817	2.817	0.370	0.545
Narrative style	Subjective cognitive load	1.350	1.350	0.177	0.675
Space capability ^*^ narrative style	Subjective cognitive load	33.750	33.750	4.434	0.040
Space capability	Use brainpower	57.958	57.958	0.243	0.624
Narrative style	Use brainpower	1789.679	1789.679	7.502	0.008
Space capability ^*^ narrative style	Use brainpower	340.388	340.388	1.427	0.237

Further paired analysis and comparison of intersubjective effects found that, in terms of using brainpower, the formal style group was significantly higher than the personalized style group in the main effect of narrative style (*P* = 0.008 < 0.05), and in the interaction effect of narrative style and spatial ability, when learners were low in spatial ability, the formal style group was significantly higher than the personalized style group (*P* = 0.007 < 0.05).

(4) Attention analysis

In this study, eye movement data of subjects in the learning process were collected with an eye tracker, and interest areas were drawn in the key areas of neutral learning materials. The total fixation times and average fixation time of interest areas were taken as dependent variables, and SPSS26 was used for a two factor analysis of variance. Descriptive statistics of various data were shown in Table 9.

**Table 9:** Descriptive statistical results of learner attention.

**Attention**	**Space ability**	**Narrative style**	***N***	***M***	**SD**
Total fixation times	High spatial ability	Personalized style	15	368.733	61.628
		Formal style	15	279.867	38.641
	Low spatial ability	Personalized style	15	362.867	68.578
		Formal style	15	322.667	61.269
Average fixation duration (ms)	High spatial ability	Personalized style	15	405.480	105.010
		Formal style	15	356.900	56.845
	Low spatial ability	Personalized style	15	397.820	67.571
		Formal style	15	334.913	66.332
Concentration	High spatial ability	Personalized style	15	51.087	16.891
		Formal style	15	44.928	16.283
	Low spatial ability	Personalized style	15	53.885	16.233
		Formal style	15	38.198	11.845

In terms of total fixation times, the main effect of spatial ability was not significant (*F* = 1.488, *P* = 0.228 > 0.05), the main effect of narrative style was significant (*F* = 18.175, *P* = 0.000 < 0.05), and the interaction effect of spatial ability and narrative style was not significant (*F* = 2.584, *P* = 0.114 > 0.05), as shown in Table 10.

**Table 10:** Results of variance analysis of total fixation times.

**Independent variable**	**Dependent variable**	**Sum of squares**	**Mean square**	***F***	**Significance**
Space capability	Total fixation times	5,115.267	5,115.267	1.488	0.228
Narrative style	Total fixation times	62,468.267	62,468.267	18.175	0.000
Space capability ^*^ narrative style	Total fixation times	8,881.667	8,881.667	2.584	0.114

Further paired analysis and comparison of intersubjective effects found that the main effect of narrative style in the personalized style group was significantly higher than that in the formal style group (*P* = 0.000 < 0.05). In the interaction effect of narrative style and spatial ability, when learners were high spatial ability, the personalized style group was significantly higher than the formal style group (*P* = 0.000 < 0.05).

In terms of average fixation time, the main effect of spatial ability was not significant (*F* = 0.568, *P* = 0.454 > 0.05), the main effect of narrative style was significant (*F* = 8.028, *P* = 0.006 < 0.05), and the interaction effect of spatial ability and narrative style was not significant (*F* = 0.133, *P* = 0.717 > 0.05), as shown in Table 11.

**Table 11:** Results of variance analysis of mean fixation time.

**Independent variable**	**Dependent variable**	**Sum of squares**	**Mean square**	***F***	**Significance**
Space capability	Average fixation duration	3,295.968	3,295.968	0.568	0.454
Narrative style	Average fixation duration	46,609.788	46,609.788	8.028	0.006
Space capability ^*^ narrative style	Average fixation duration	769.700	769.700	0.133	0.717

Further paired analysis and comparison of intersubjective effects found that the main effect of narrative style in the personalized style group was significantly higher than that in the formal style group (*P* = 0.006 < 0.05). In the interaction effect of narrative style and spatial ability, when learners were low spatial ability, the personalized style group was significantly higher than the formal style group (*P* = 0.028 < 0.05).

In terms of focus, the main effect of spatial ability was not significant (*F* = 0.243, *P* = 0.624 > 0.05), the main effect of narrative style was significant (*F* = 7.502, *P* = 0.008 < 0.05), and the interaction effect of spatial ability and narrative style was not significant (*F* = 1.427, *P* = 0.237 > 0.05), as shown in Table 12.

**Table 12:** Analysis results of concentration variance.

**Independent variable**	**Dependent variable**	**Sum of squares**	**Mean square**	***F***	**Significance**
Space capability	Concentration	57.958	57.958	0.243	0.624
Narrative style	Concentration	1,789.679	1,789.679	7.502	0.008
Space capability ^*^ narrative style	Concentration	340.388	340.388	1.427	0.237

Further paired analysis and comparison of intersubjective effects found that the main effect of narrative style in the personalized style group was significantly higher than that in the formal style group (*P* = 0.008 < 0.05). In the interaction effect of narrative style and spatial ability, when learners were low spatial ability, the personalized style group was significantly higher than the formal style group (*P* = 0.007 < 0.05).

### 4.2 Experiment 2: exploring the impact of the personalization principle on learning outcomes in negative materials

(1) Experimental hypothesis

This experiment mainly discusses the influence of personalization principle on the learning effect of learners with different spatial abilities in negative learning materials. The independent variable of the experiment is 2 (high and low spatial abilities) × 2 (personalized style, formal style). The dependent variable of the experiment is academic achievement, cognitive load, attention and emotion.

The influence of narrative style on learners' academic performance, cognitive load, attention and emotion in augmented reality environment:

H1a There are significant differences in learners' performance under the influence of different narrative styles, and the formal style is significantly higher than the personalized style.
H1b Learners' transfer performance is significantly different under the influence of different narrative styles, and the formal style is significantly higher than the personalized style.
H2a Learners' cognitive load is significantly different under the influence of different narrative styles, and the formal style is significantly lower than the personalized style.

The positive emotions of formal style are significantly higher than personalized style, and the negative emotions of formal style are significantly lower than personalized style.

H3 Learners' attention is significantly different under the influence of different narrative styles, and the attention of formal style is significantly higher than that of personalized style.

The effects of learners' spatial ability on academic performance, cognitive load, attention and emotion in augmented reality environment:

H4a Learners with different spatial abilities have significant differences in retention performance, and learners with high spatial abilities are significantly higher than those with low spatial abilities.
H4b Learners with different spatial abilities have significant differences in transfer performance, and learners with high spatial abilities are significantly higher than those with low spatial abilities.
H5a Learners with different spatial abilities have significant differences in cognitive load, and learners with high spatial abilities are significantly lower than those with low spatial abilities.
H5b Learners with different spatial abilities have significant emotional differences. The positive emotions of high spatial ability learners are significantly higher than those of low spatial ability learners, and the negative emotions of high spatial ability learners are significantly lower than those of low spatial ability learners.
H6 The attention of learners with different spatial abilities is significantly different, and the attention of learners with high spatial abilities is significantly higher than that of learners with low spatial abilities.

(2) Analysis of academic achievements

In this study, the retention and transfer scores of the subjects are taken as dependent variables, the spatial ability of learners and the narrative style of learning materials are taken as independent variables, and the previous test scores are taken as covariates. SPSS26 is used for a two factor analysis of variance. Descriptive statistics of various data are shown in Table 13.

**Table 13:** Descriptive statistical results of learners' academic achievements.

**Score type**	**Space capability**	**Narrative style**	***N***	***M***	**SD**
Maintain performance	High space capability	Personalized style	15	9.13	2.996
		Formal Style	15	9.67	2.919
	Low space capability	Personalized style	15	7.07	2.548
		Formal Style	15	7.67	2.743
Migration achievements	High space capability	Personalized style	15	3.20	1.082
		Formal Style	15	3.33	1.234
	Low space capability	Personalized style	15	2.53	0.743
		Formal style	15	2.60	0.910
Prior knowledge	High space capability	Personalized style	15	3.60	1.121
		Formal Style	15	3.80	1.082
	Low space capability	Personalized style	15	3.53	0.833
		Formal style	15	3.47	1.187

In order to explore whether there are differences in the level of prior knowledge among the four groups of subjects, this experiment compares the scores of prior knowledge of the four groups of learners as a dependent variable. The results of one-way ANOVA show that there is no significant difference in the prior knowledge of the four groups of subjects (*F* = 0.275, *P* = 0.844 > 0.05), as shown in Table 14. Therefore, there is no significant difference in the level of prior knowledge among the four groups of learners, and the experiment can be carried out.

**Table 14:** Results of variance analysis of learners' prior knowledge.

**Dependent variable**	**Sum of squares**	**Mean square**	***F***	**Significance**
Prior knowledge	0.933	0.311	0.275	0.844

The post test learning achievement includes learning retention achievement and transfer achievement. The two factor analysis of variance found that in terms of retention achievement, the main effect of spatial ability was significant (*F* = 7.869, *P* = 0.007 < 0.05), the main effect of narrative style was not significant (*F* = 0.611, *P* = 0.438 > 0.05), and the interaction effect of spatial ability and narrative style was not significant (*F* = 0.002, *P* = 0.963 > 0.05); In terms of transfer performance, the main effect of spatial ability was significant (*F* = 7.213, *P* = 0.01 < 0.05), the main effect of narrative style was not significant (*F* = 0.147, *P* = 0.703 > 0.05), and the interaction effect of spatial ability and narrative style was not significant (*F* = 0.016, *P* = 0.899 > 0.05), as shown in Table 15.

**Table 15:** Results of variance analysis of learners' posttest scores.

**Independent variable**	**Dependent variable**	**Sum of squares**	**Mean square**	***F***	**Significance**
Space capability	Maintain performance	62.017	62.017	7.869	0.007
	Migration achievements	7.350	7.350	7.213	0.01
Narrative style	Maintain performance	4.817	4.817	0.611	0.438
	Migration achievements	0.150	0.150	0.147	0.703
Space capability ^*^ narrative style	Maintain performance	0.017	0.017	0.002	0.963
	Migration achievements	0.017	0.017	0.016	0.899

Further paired comparative analysis of inter subject effects showed that, in terms of maintaining performance, the main effects of high spatial ability group were significantly higher than those of low spatial ability group (*P* = 0.007 < 0.05); In the interaction effect between spatial ability and narrative style, when the narrative style was formal, the high spatial ability group was significantly higher than the low spatial ability group (*P* = 0.049 < 0.05). In terms of transfer performance, in the main effect of spatial ability, the high spatial ability group was significantly higher than the low spatial ability group (*P* = 0.01 < 0.05).

(3) Learning experience analysis

In this study, the subjective questionnaire of cognitive load, the emotional questionnaire and the degree of brain use and relaxation measured by the brain wave instrument filled in by the subjects were taken as the dependent variables, and the learners' spatial ability and learning material presentation style were taken as the independent variables. Spss26 was used for two-way ANOVA. The descriptive statistics of the data are shown in Table 16.

**Table 16:** Descriptive statistical results of learning experience.

**Dependent variable**	**Space capability**	**Narrative style**	***N***	***M***	**SD**
Subjective cognitive load	High space capability	Personalized style	15	11.07	2.282
		Formal style	15	11.07	2.520
	Low space capability	Personalized style	15	11.00	2.390
		Formal style	15	10.33	1.633
Use brainpower	High space capability	Personalized style	15	38.78	9.392
		Formal style	15	44.82	11.672
	Low space capability	Personalized style	15	43.93	10.476
		Formal Style	15	44.29	7.414
Previous positive emotions	High space capability	Personalized style	15	23.93	3.673
		Formal style	15	32.06	9.05
	Low space capability	Personalized style	15	25.13	8.568
		Formal style	15	25.47	7.781
Previous negative emotions	High space capability	Personalized style	15	25.60	8.122
		Formal style	15	19.27	8.971
	Low space capability	Personalized style	15	22.47	7.308
		Formal style	15	18.60	10.133
Positive emotions	High space capability	Personalized style	15	28.20	4.003
		Formal style	15	37.07	7.995
	Low space capability	Personalized style	15	28.73	8.362
		Formal style	15	32.20	8.046
Negative emotions	High space capability	Personalized style	15	19.87	8.096
		Formal style	15	13.73	6.318
	Low space capability	Personalized style	15	19.73	9.075
		Formal style	15	12.47	6.457
Relaxation	High space capability	Personalized style	15	55.06	8.372
		Formal style	15	57.03	7.975
	Low space capability	Personalized style	15	54.72	10.890
		Formal style	15	55.9	11.449

Cognitive load includes subjective cognitive load and objectively measured brain use data. Two factor analysis of variance found that in terms of subjective cognitive load, the main effect of spatial ability was not significant (*F* = 0.481, *P* = 0.491 > 0.05), the main effect of narrative style was not significant (*F* = 0.334, *P* = 0.565 > 0.05), and the interaction effect of spatial ability and narrative style was not significant (*F* = 0.334, *P* = 0.565 > 0.05); In terms of brain use, the main effect of spatial ability was not significant (*f* = 0.826, *p* = 0.367 > 0.05), the main effect of narrative style was not significant (*f* = 1.578, *p* = 0.214 > 0.05), and the interaction effect of spatial ability and narrative style was not significant (*f* = 1.247, *p* = 0.269 > 0.05), as shown in Table 17.

**Table 17:** Results of variance analysis of cognitive load.

**Independent variable**	**Dependent variable**	**Sum of squares**	**Mean square**	***F***	**Significance**
Space capability	Subjective cognitive load	2.400	2.400	0.481	0.491
Narrative style	Subjective cognitive load	1.667	1.667	0.334	0.565
Space capability ^*^ narrative style	Subjective cognitive load	1.667	1.667	0.334	0.565
Space capability	Use brainpower	80.342	80.342	0.826	0.367
Narrative style	Use brainpower	153.504	153.504	1.578	0.214
Space capability ^*^ narrative style	Use brainpower	121.297	121.297	1.247	0.269

In order to explore whether there is a difference in the level of prior positive emotions and negative emotions among the four groups of subjects, this experiment compared the previous emotions of the four groups of learners as the dependent variable. The results of one-way ANOVA showed that there was no significant difference in the previous positive emotions of the four groups of subjects (*f* = 0.336, *p* = 0.799 > 0.05), and there was no significant difference in the previous negative emotions (*f* = 2.058, *p* = 0.116 > 0.05), as shown in Table 18. Therefore, the four groups of learners have no significant difference in the previous emotional level, so the experiment can be carried out.

**Table 18:** Results of variance analysis of learners' previous emotions.

**Dependent variable**	**Sum of squares**	**Mean square**	***F***	**Significance**
Previous positive emotions	18.733	6.244	0.336	0.799
Previous positive emotions	467.117	155.706	2.058	0.116

The two-way ANOVA of the post-test positive emotion level and negative emotion level found that in terms of positive emotion, the main effect of spatial ability was not significant (*f* = 1.312, *p* = 0.257 > 0.05), the main effect of narrative style was significant (*f* = 10.631, *p* = 0.002 < 0.05), and the interaction effect of spatial ability and narrative style was not significant (*f* = 2.038, *p* = 0.159 > 0.05). In terms of negative emotions, the main effect of spatial ability was not significant (*f* = 0.128, *p* = 0.722 > 0.05), the main effect of narrative style was significant (*f* = 11.735, *p* = 0.00 < 0.05), and the interaction effect of spatial ability and narrative style was not significant (*f* = 0.084, *p* = 0.773 > 0.05). In terms of brain use, the main effect of spatial ability is not significant (*f* = 0.083, *p* = 0.774 > 0.05), the main effect of narrative style is significant (*f* = 0.387, *p* = 0.536 > 0.05), and the interaction effect of spatial ability and narrative style is not significant (*f* = 0.025, *p* = 0.876 > 0.05), as shown in Table 19.

**Table 19:** Results of variance analysis of posttest emotion of learners.

**Independent variable**	**Dependent variable**	**Sum of squares**	**Mean square**	***F***	**Significance**
Space capability	Positive emotions	70.417	70.417	1.312	0.257
	Negative emotions	7.350	7.350	0.128	0.722
	Relaxation	7.964	7.964	0.083	0.774
Narrative style	Positive emotions	570.417	570.417	10.631	0.002
	Negative emotions	673.350	673.350	11.735	0.001
	Relaxation	37.131	37.131	0.387	0.536
Space capability ^*^ narrative style	Positive emotions	109.350	109.350	2.038	0.159
	Negative emotions	4.817	4.817	0.084	0.773
	Relaxation	2.368	2.368	0.025	0.876

Further paired comparative analysis of the intersubjective effects of positive emotions found that the formal style group was significantly higher than the personalized style group in the main effect of narrative style (*p* = 0.002 < 0.05). In the interaction effect of narrative style and spatial ability, when the learner was high spatial ability, the formal style group was significantly higher than the personalized style group (*p* = 0.002 < 0.05).

Further paired comparative analysis of the inter subjective effects of negative emotions found that the main effect of narrative style in the personalized style group was significantly higher than that in the formal style group (*p* = 0.001 < 0.05). In the interactive effect of narrative style and spatial ability, when the learner was low spatial ability, the personalized style group was significantly higher than that in the formal style group (*p* = 0.011 < 0.05). When the learner was high spatial ability, the personalized style group was significantly higher than that in the formal style group (*p* = 0.031 < 0.05).

(4) Attention analysis

In this study, eye movement data of subjects during learning were collected with an eye tracker, and interest areas were drawn in key areas of negative learning materials. The total fixation times and average fixation time of interest areas were taken as dependent variables, and SPSS26 was used for a two factor analysis of variance. Descriptive statistics of various data were shown in Table 20.

**Table 20:** Descriptive statistical results of learner attention.

**Attention**	**Space capability**	**Narrative style**	***N***	***M***	**SD**
Total fixation times	High space capability	Personalized style	15	303.0667	82.884
		Formal style	15	375.800	95.387
	Low space capability	Personalized style	15	328.800	58.172
		Formal style	15	362.400	99.456
Average fixation duration (ms)	High space capability	Personalized style	15	337.8733	109.033
		Formal style	15	373.120	60.248
	Low space capability	Personalized style	15	328.4467	48.458
		Formal style	15	388.693	41.476
Concentration	High space capability	Personalized style	15	52.421	4.569
		Formal style	15	58.843	13.914
	Low space capability	Personalized style	15	54.210	4.240
		Formal style	15	59.092	11.317

In terms of total fixation times, the main effect of spatial ability was not significant (*f* = 0.078, *p* = 0.781 > 0.05), the main effect of narrative style was significant (*f* = 5.799, *p* = 0.019 < 0.05), and the interaction effect of spatial ability and narrative style was not significant (*f* = 0.785, *p* = 0.379 > 0.05), as shown in Table 21.

**Table 21:** Results of variance analysis of total fixation times.

**Independent variable**	**Dependent variable**	**Sum of squares**	**Mean square**	***F***	**Significance**
Space capability	Total fixation times	570.417	570.417	0.078	0.781
Narrative style	Total fixation times	42,400.417	42,400.417	5.799	0.019
Space capability ^*^ narrative style	Total fixation times	5,742.817	5,742.817	0.785	0.379

Further paired analysis and comparison of inter subject effects showed that the main effect of narrative style in the formal style group was significantly higher than that in the personalized style group (*p* = 0.019 < 0.05), and the interaction effect between narrative style and spatial ability in the formal style group was significantly higher than that in the personalized style group (*p* = 0.023 < 0.05).

In terms of average fixation time, the main effect of spatial ability was not significant (*f* = 0.029, *p* = 0.866 > 0.05), the main effect of narrative style was significant (*f* = 6.984, *p* = 0.011 < 0.05), and the interaction effect of spatial ability and narrative style was not significant (*f* = 0.479, *p* = 0.492 > 0.05), as shown in Table 22.

**Table 22:** Results of variance analysis of mean fixation time.

**Independent variable**	**Dependent variable**	**Sum of squares**	**Mean square**	***F***	**Significance**
Space capability	Average fixation duration	141.681	141.681	0.029	0.866
Narrative style	Average fixation duration	34,196.163	34,196.163	6.984	0.011
Space capability ^*^ narrative style	Average fixation duration	2,343.750	2,343.750	0.479	0.492

Further paired analysis and comparison of inter subject effects showed that the formal style group was significantly higher than the personalized style group in the main effect of narrative style (*p* = 0.011 < 0.05), and the formal style group was significantly higher than the personalized style group in the interaction effect of narrative style and spatial ability when the learner was low spatial ability (*p* = 0.022 < 0.05).

In terms of concentration, the main effect of spatial ability is not significant (*f* = 0.173, *p* = 0.679 > 0.05), the main effect of narrative style is significant (*f* = 5.317, *p* = 0.025 < 0.05), and the interaction effect of spatial ability and narrative style is not significant (*f* = 0.099, *p* = 0.755 > 0.05), as shown in Table 23.

**Table 23:** Analysis results of concentration variance.

**Independent variable**	**Dependent variable**	**Sum of squares**	**Mean square**	***F***	**Significance**
Space capability	Concentration	15.575	15.575	0.173	0.679
Narrative Style	Concentration	479.233	479.233	5.317	0.025
Space capability ^*^ narrative style	Concentration	8.901	8.901	0.099	0.755

Further pairwise analysis and comparison of inter subject effects showed that the main effect of narrative style in the formal style group was significantly higher than that in the personalized style group (*p* = 0.025 < 0.05).

## 5 Conclusion and discussion

### 5.1 Experimental conclusion of the influence of personalization principle on learning effect

(1) Conclusion on the influence of individualization principle in neutral materials on learning effect

In terms of academic performance, personalized narrative style can significantly improve the retention performance of low spatial ability learners and the transfer performance of high spatial ability learners; Learners' high and low spatial abilities will not affect their academic performance. In terms of cognitive load, narrative style and spatial ability have no significant impact, but when learners have low spatial ability, formal style narrative learning content will significantly increase learners' subjective psychological effort and brain use. In terms of attention, narrative style will have a significant impact on learners' attention, and learners' attention in personalized style is significantly higher than that in formal style group; When learners have low spatial ability, learning materials described in a personalized style can increase their attention.

(2) Conclusion of the influence of individualization principle on learning effect in negative materials

In terms of academic performance, the retention and transfer performance of learners in the formal style group were higher than those in the personalized style group, but the difference was not significant; High spatial ability learners perform better in retention and transfer performance. When speaking in a formal style, the learning performance of high spatial ability learners is significantly higher than that of low spatial ability learners. In terms of cognitive load, narrative style and spatial ability had no significant impact. In terms of emotion, formal style narration can stimulate learners' positive emotions and reduce learners' negative emotions better than personalized style narration. In terms of attention, narrative style has a significant impact on learners' attention, and learners' attention in formal style is significantly higher than that in personalized style group.

### 5.2 Discussion on the experimental results of the influence of personalization principle on learning effect

(1) The influence of individualization principle in neutral materials on academic performance

As for the effect of narrative style, hypothesis H1a and H1b are true in Experiment 1. In neutral learning materials, personalized narrative style can significantly improve learners' retention and transfer performance. This finding is consistent with the previous research results in the traditional learning environment, which shows that the personalized principle does not become inapplicable due to the change of the learning environment. Mayer (2014) takes the personalized narrative style as a social clue according to the social agency theory, which will encourage them to participate in Generative processing and produce more meaningful learning outcomes (Mayer, 2014). Personalized narrative style can significantly improve the performance of learners with low spatial ability. It may be the personalized narrative style learning materials presented in three-dimensional way in the AR learning environment, creating a sense of dialogue with teachers, encouraging learners to engage in the cognitive processing required for meaningful learning, helping them better understand and remember knowledge, so as to improve the performance. Personalized narrative style significantly improves the transfer performance of high spatial ability learners. It may be that high spatial ability learners have strong spatial cognition and imagination ability, have advantages in processing visual and spatial information, and can effectively integrate and operate complex spatial information (Hegarty and Waller, 2005), personalized narrative style further pulls the distance between learners and learning content, which helps learners connect new and old knowledge, thus promoting knowledge transfer.

In terms of the effect of spatial capability, the hypothesis h4a and H4b in Experiment 1 is not tenable. The results show that spatial ability does not have a significant impact on academic performance. This result is inconsistent with some research conclusions. The possible reason is that the learning material used in this experiment is “the structure of the heart”, and does not involve tasks that are highly dependent on spatial ability (such as geometry and 3D folding). The influence of spatial ability may be masked by the characteristics of learning materials. In addition, the personalized narrative style may balance the differences between high and low spatial ability learners in the learning process to a certain extent, thus weakening the direct effect of spatial ability on academic performance.

(2) The influence of personalization principle on cognitive load in neutral materials

For the effect of narrative style, the hypothesis H2 in Experiment 1 is not tenable. The experimental results show that the use of different narrative styles in neutral materials has no significant effect on learners' cognitive load, but the formal style of narrative learning content will significantly increase learners' subjective psychological effort and brain use in low spatial ability. According to the cognitive load theory, when the presentation of learning materials does not match learners' cognitive ability, it will increase their external cognitive load and lead to higher psychological effort (Sweller, 1988). The reason may be that the formal style explanation puts forward higher cognitive requirements for learners with low spatial ability, which leads them to need to invest more psychological resources and mental activities in understanding and processing information.

For the effect of spatial capability, the hypothesis H5 in Experiment 1 is not tenable. The three-dimensional 3D model and animation demonstration in AR learning environment may make up for the cognitive limitations of low spatial ability learners to a certain extent, thus reducing the impact of spatial ability differences on cognitive load.

(3) The influence of individuation principle on attention in neutral materials

For the effect of narrative style, hypothesis H3 of Experiment 1 is true. The use of personalized narrative style can significantly improve learners' fixation times and average fixation time for the area of interest. This is consistent with the research results of Zander et al. (2015), whose eye tracking data shows that learners show longer fixation duration and more fixation count when contacting personalized content, indicating that personalized content can effectively attract and maintain learners' attention. Personalized narrative style makes learners feel that the content is related to themselves, and the learning content is related to their own organs. This kind of knowledge content familiar to learners can enhance attraction, reduce distractions, and focus learners' attention. On the contrary, the formal style usually uses abstract language and does not add direct language guidance to learners, resulting in a lack of resonance between learners and learning content, and it is difficult to maintain attention.

For the effect of spatial capability, the hypothesis H6 in Experiment 1 is not tenable. Learners' cognitive style may regulate the relationship between spatial ability and attention. Some learners may be more inclined to visual cognitive style, and can make better use of spatial ability to improve attention when processing spatial information (Zander et al., 2015). However, for non-visual cognitive style learners, their spatial ability may not significantly promote attention.

(4) The influence of individualization principle in negative materials on academic performance

As for the effect of narrative style, hypothesis H1a and H1b in Experiment 2 are not tenable. The experimental results show that learners' performance in knowledge retention and transfer in the formal style group is slightly higher than that in the personalized style group, but the difference is not significant. Kühl and Zander (2017) found that in the learning of negative content, personalized design does not improve the learning effect, but may reduce the performance of learners. The reason why this study does not have the inversion effect may be that AR technology has a positive role in promoting learning effectiveness (Wan, 2021). AR technology provides learners with a highly immersive environment in which learners may separate virtual content from real emotions, reducing negative emotional reactions to negative materials. However, the descriptive results showed that the learning performance of personalized style learners was lower than that of the formal style group, which to some extent indicated that it would be better to use the formal style to narrate the negative materials. In the AR learning environment, the personalized principle may be weaker than the influence of AR technology on learners. Therefore, it is worth further exploring the conditions for the inversion effect of personalized principles in the AR learning environment in the future.

As for the effect of spatial capability, the hypothesis H4a and H4b in Experiment 2 is true. Learners with strong spatial ability are better at dealing with complex visual and spatial information, which makes them perform better in learning negative materials involving three-dimensional models. Negative materials usually contain more emotional and cognitive loads, which require learners to have strong information processing ability. High spatial ability learning can manage these loads well, while low spatial ability learners may be more vulnerable to emotional factors, thus affecting their retention and transfer performance.

(5) The influence of personalization principle on cognitive load in negative materials

As for the effect of narrative style, experiment 2 assumed that H2a was not true and H2b was true. In AR environment, learners need to process multiple sensory information (visual, auditory and tactile) at the same time, which may reduce their attention allocation to personalized design, thus reducing the impact of personalized design on cognitive load. Research shows that learners do not want to bring themselves into the learning materials to combine with negative learning content. Using personalized style to tell negative materials will increase learners' negative emotions. By using “you”, “yours” and other ways, learners are more likely to have emotional resonance with the learning content. According to the self reference effect, personalized design enables learners to connect learning content with themselves, which may make learners more likely to feel negative emotions. In the negative materials, the seriousness and importance of the content are highlighted through the formal style of narration, so that learners can be more focused on learning and understanding.

As for the effect of spatial capability, experiment 2 assumed that H5a was not tenable and H5b was partially tenable. High spatial ability learners have higher interest in this learning may be that they usually have higher cognitive needs and like to challenge complex tasks. Ar environment satisfies its cognitive needs by providing rich visual and spatial information, so as to stimulate interest.

(6) The influence of personalization principle on attention in negative materials

For the effect of narrative style, hypothesis H3 is true in Experiment 2. Negative materials are often accompanied by disgusting negative emotions. The formal style of narration usually presents the content in a relatively neutral manner, forming a certain buffer between learners and negative materials, reducing the direct impact of negative emotions, making it easier for them to bear and process these information, so as to maintain good attention. Because the subjects do not have medical background, they usually learn relevant knowledge in the form of short videos or academic lectures. This method mostly adopts the formal style of narration, which will form a stereotype psychologically. They believe that the formal style of narration is the formal learning. At the same time, the formal style of narration in the AR environment will trigger their cognitive mode of learning, making it easier for them to enter the learning state and concentrate.

For the effect of spatial capability, hypothesis H6 in Experiment 2 is not tenable. The knowledge of negative materials is mainly about the location and symptoms of cerebral hemorrhage. This disease occurs from time to time in life and can arouse the curiosity and attention of learners. Its novelty is enough to attract learners' attention, making the role of spatial ability and other factors in attention distribution relatively less prominent.

### 5.3 Countermeasures and suggestions

The purpose of this study is to explore whether the principle of personalization can be transferred to AR learning environment, and to verify the effectiveness of the principle of personalization through different kinds of learning materials.

(1) Strategies for neutral learning materials

In neutral materials, personalized storytelling style can effectively improve learners' retention and transfer performance, especially for learners with low spatial abilities. Therefore, it is recommended to prioritize personalized storytelling styles in the study of neutral materials, especially for learners with low spatial abilities. Personalized storytelling style can significantly enhance learners' attention, especially those with low spatial abilities. When narrating or explaining neutral learning materials, first and second person can be used to enhance learners' attention. Although narrative style and spatial ability do not have a significant impact on cognitive load, learners with low spatial ability will feel higher psychological effort and brainpower under formal style. Therefore, for learners with low spatial abilities, overly formal narrative styles should be avoided to alleviate their cognitive burden.

(2) Strategies for Negative Learning Materials

In negative materials, formal narrative style can enhance learners' retention and transfer performance, especially for learners with high spatial abilities. Therefore, in the study of negative materials, it is recommended to adopt a formal narrative style, especially for learners with high spatial abilities. Formal narrative style can stimulate the interest and value of high spatial ability learners. Therefore, in the learning of negative materials, formal narrative style can be used to enhance learners' intrinsic motivation, especially for learners with high spatial abilities. Formal narrative style can also stimulate learners' positive emotions and reduce negative emotions. Therefore, in the study of negative materials, adopting a formal narrative style can help create a positive learning atmosphere and enhance learners' emotional state. In negative materials, formal narrative style can significantly enhance learners' attention. Therefore, when narrating or explaining negative materials, a formal narrative style can be used to enhance learners' attention and avoid bringing them into the learning context.

## 6 Summary and prospect

### 6.1 Research summary

Through the development of two ar educational resources, this study systematically discusses the influence of personalized language style in different materials on learners' learning effect from the thematic nature of learning materials (neutral and negative) and learners' characteristics (high and low spatial ability). Using a quasi experimental design, combined with eye tracking technology and brain wave technology, we comprehensively investigated the multi-dimensional data of learners' attention distribution, cognitive load, emotional state and academic performance in different materials. Research shows that personalized language style can significantly improve the performance and attention of low spatial ability learners in neutral materials, while formal language style is more conducive to the learning performance and positive emotions of high spatial ability learners in negative materials. In addition, the study shows that the interaction between narrative style and spatial ability shows significant differences in different materials, and the applicability of the principle of personalization depends on the matching of material properties and learners' characteristics.

This study provides an important theoretical basis and practical guidance for the design and development of AR educational resources, defines the advantages of personalized language style in neutral materials, reveals the applicability of formal language style in negative materials, and enriches the multidimensional perspectives of learners' attention distribution, cognitive load and emotional state assessment. Future research can further expand the range of material types and learners' characteristics, and explore the application effect of personalization principle in more situations, so as to promote the wider application and optimization of AR technology in the field of education.

### 6.2 Research shortcomings and prospects

(1) Limitations of material type

This study only divides learning materials into neutral and negative categories, and does not cover more types of materials. The universality of research conclusions may be limited. In the future, the applicability of personalization principle can be further discussed in active materials and mixed materials.

(2) Uniqueness of learner characteristics

The study only takes spatial ability as the basis for the division of learner characteristics, and does not consider other factors that may affect the learning effect, such as learning style, prior knowledge level, motivation type, etc. The lack of these factors may limit the comprehensiveness and pertinence of the research conclusions.

(3) Insufficient sample size and diversity

The sample size of the study may be small, and the subjects are college students. The gender ratio of the subjects is unbalanced, and factors such as school period, cultural background, education level, etc. are not fully considered. It may lead to the limitation of its popularization and its inability to fully represent a broader group of learners.

(4) Limitations of the experimental environment

The research was conducted in an augmented reality (AR) environment, but it was not compared with other learning environments (such as traditional classroom, online learning, etc.). Therefore, it is impossible to determine the unique contribution of the AR environment itself to the learning effect, nor to assess the applicability of the personalization principle in other environments. In the future, resources with the same content as AR education resources can be designed to compare with traditional classroom teaching.

(5) Lack of long-term effect

The research mainly focuses on short-term learning effects (such as retention and transfer), and does not examine the impact of personalization principle on long-term learning effects of learners. This limits the assessment of the long-term value of the personalization principle.

## Data Availability

The raw data supporting the conclusions of this article will be made available by the authors, without undue reservation.
