# Supplementary material for: Research on the influence of personalized principles in AR educational resources on the learning effectiveness of college students
Source: Front Psychol. 2026 Jan 14;16:1618990. doi: 10.3389/fpsyg.2025.1618990 (PMC12848803; doi:10.3389/fpsyg.2025.1618990)
Supplement: Supplementary file 1 [file Supplementary_file_1.doc]

## Appendix1 A priori knowledge questionnaire

Experiment 1 A priori knowledge questionnaire：

| **Title No** | **Item** | **Very little understanding**    **Very familiar** | | | | | | |
| --- | --- | --- | --- | --- | --- | --- | --- | --- |
|  |  | **1** | **2** | **3** | **4** | **5** | **6** | **7** |
| 1 | Your understanding of blood composition |  |  |  |  |  |  |  |
| 2 | Your understanding of human heart structure |  |  |  |  |  |  |  |
| 3 | Your understanding of human blood circulation |  |  |  |  |  |  |  |

Experiment 2A priori knowledge questionnaire：

| **Title No** | **Item** | **Very little understanding**    **Very familiar** | | | | | | |
| --- | --- | --- | --- | --- | --- | --- | --- | --- |
|  |  | **1** | **2** | **3** | **4** | **5** | **6** | **7** |
| 1 | Your understanding of human brain structure |  |  |  |  |  |  |  |
| 2 | Your understanding of cerebral hemorrhage |  |  |  |  |  |  |  |

## Appendix2 Space ability test questionnaire

1、You can imagine a piece of paper folded along the dotted line, and then make a hole through the folded paper. Please select the image after opening the paper from the following five options.


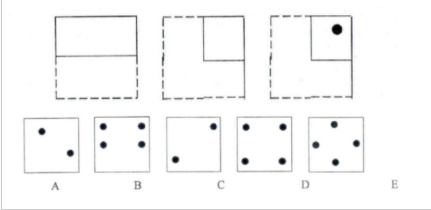
2、You can imagine a piece of paper folded along the dotted line, and then make a hole through the folded paper. Please select the image after opening the paper from the following five options.


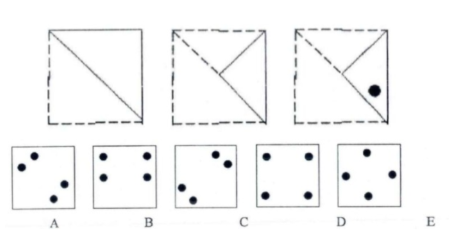


3、According to the given figure, you need to select the same one from the following four figures, allowing rotation of various angles.


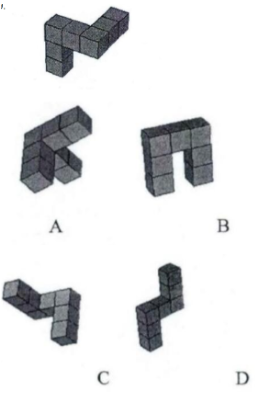


4、


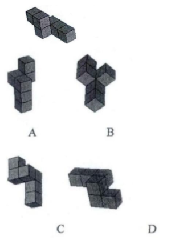


5、


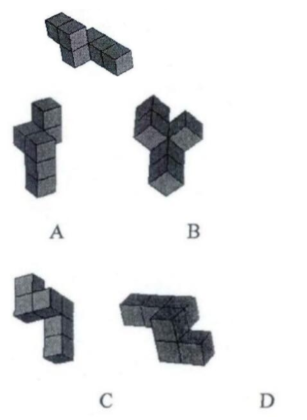


6、


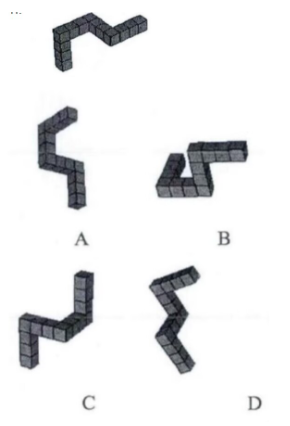


7、


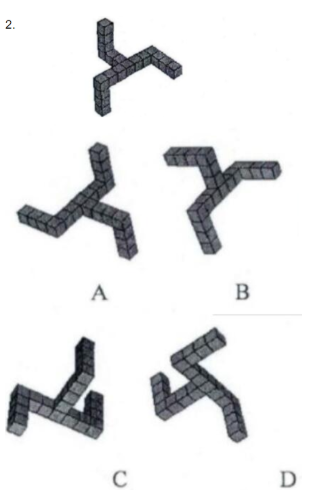


8、


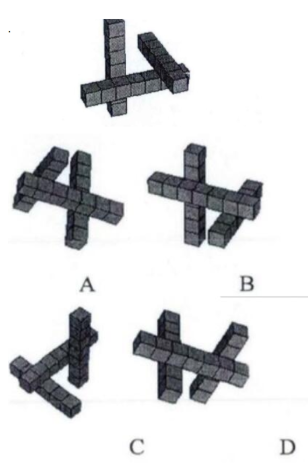


9、


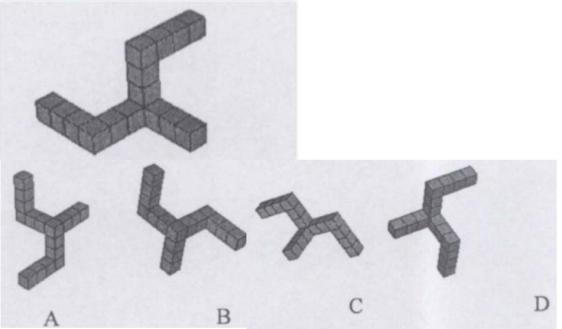


10、


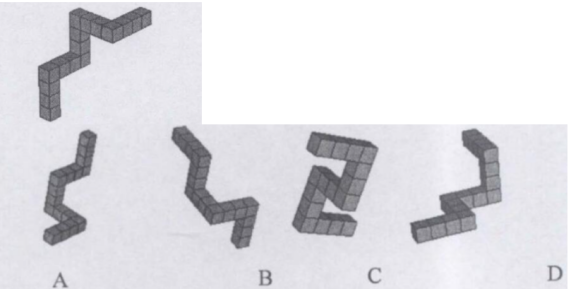


## Appendix3 Academic Achievement Measurement Questionnaire

**Experiment 1: Measurement of retention performance:**

1. What are the components of blood?

A. Plasma and blood cells

B. Plasma and red blood cells

C. Plasma and leukocytes

D. Platelets

2. Why is blood red?

A. Because red blood cells are red.

B. Because red is the color of plasma

C. Because red is the color of hypoxic blood

D. Because oxygen makes it red

3. How many chambers does the human heart have?

A. 2; 1 atrium, 1 ventricle

B. 3; 1 atrium, 1 left ventricle and 1 right ventricle

C. 4; 1 upper ventricle and 1 lower ventricle, 1 upper atrium and 1 lower atrium

D. 4; 1 right ventricle and 1 left ventricle, 1 right atrium and 1 left atrium

4. Which of the following is most similar to the heart?

A. Hose, because blood flows in the pipe.

B. The cup, because its top is open.

C. Broom, because it can remove blood stains.

D. Pump, because it pushes blood into the body.

5. In the pulmonary circulation, the right ventricle pumps blood to

A. Left atrium B. Right atrium C. Left ventricle D. Lung

6. What is the blood route in the pulmonary circulation?

A. Heart ->Lung ->Heart

B. Lung ->Heart ->Lung

C. Liver ->Lung ->Heart

D. Left ventricle ->left atrium ->right atrium

7. In systemic circulation, where does blood finally gather?

A. Right ventricle. B. Right atrium. C. Left ventricle. D. Left atrium.

8. When blood flows from the heart to the body through systemic circulation, which part of the heart will it leave?

A. Left atrium. B. Right ventricle. C. Left ventricle. D. Right atrium. Judgmental question

9. The function of plasma is mainly to transport the substances needed for human life. A. Correct B. Error

10. White blood cells have no nuclei.

A. Correct B. Error

11. The left ventricle is connected to the aorta.

A. Correct B. Error

12. Right atrium is connected with the superior vena cava and inferior vena cava.

A. Correct B. Error

Completion

The figure is the schematic diagram of human blood circulation system. Please answer according to the figure


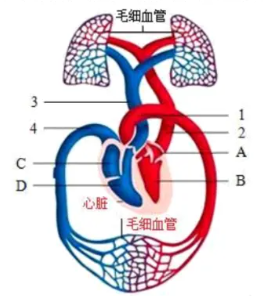


1. A is:
2. D is:
3. 1 Yes:

16. 3 Yes:

**Experiment 1 Transfer performance measurement:**

1. Why is the heart the most important organ of the human body?

2. Why does exercise help blood circulation?

**Experiment 2: Measurement of retention performance:**

1. What are the main parts of the brain?

A-end brain, cerebellum and diencephalon

B Brain stem, cerebellum and cerebral cortex

C diencephalon, brain stem and cerebellum

D-terminal brain, diencephalon and cerebral cortex

2. Does the main function of cerebellum include?

A Muscle coordination B Balance C Memory D Motor skills

3. Which of the following is not a common cause of cerebral hemorrhage?

A Hypertension B Diabetes C Cerebrovascular malformation D Brain tumor

4. Common symptoms of cerebral hemorrhage do not include?

A Severe headache and disturbance of consciousness

B Nausea and vomiting

C Limb weakness

D Tinnitus

5. What is the most common cause of subarachnoid hemorrhage?

A Hypertension B Blood disease

C Rupture of cerebral aneurysm D Cerebrovascular malformation

6. Where is subarachnoid hemorrhage usually located?

A Between brain and meninges B Between dura and skull

C Between dura and arachnoid D Inside brain tissue

7. What is the most common cause of intraventricular hemorrhage?

A Hypertension B Blood disease

C Rupture of cerebral aneurysm D Cerebrovascular malformation

8. Which of the following bleeding is most likely to occur if the head is injured?

A Subarachnoid hemorrhage B intraventricular hemorrhage

C Subdural hemorrhage D Brain stem hemorrhage

9. Once cerebral hemorrhage occurs, which of the following is not a possible consequence?

A Increase the pressure on the skull

B Increased blood flow to downstream cells

Hypoxia of tissue cells near C

D Health tissue may die within hours

10. Short answer question: Please describe the names of the four main functional areas of the cerebral cortex and their main functions.

**Experiment 2 Measurement of transfer performance:**

1. Short answer question: What do you think should be used to deal with cerebral hemorrhage?

2. Short answer question: What should we do in life to reduce the risk of cerebral hemorrhage?

## Appendix4 Cognitive Load Measurement Questionnaire

A、The difficulty of the learning task just carried out

| 1 | 2 | 3 | 4 | 5 | 6 | 7 | 8 | 9 |
| --- | --- | --- | --- | --- | --- | --- | --- | --- |

Very easy Medium Very difficult

B、The degree of effort in completing the teaching video learning task

| 1 | 2 | 3 | 4 | 5 | 6 | 7 | 8 | 9 |
| --- | --- | --- | --- | --- | --- | --- | --- | --- |

## Very easy General efforts All efforts

## Appendix5 Emotional Measurement Questionnaire

| **Title No** | **Item** | Completely inconformity  Fully compliant | | | | | | |
| --- | --- | --- | --- | --- | --- | --- | --- | --- |
|  |  | 1 | 2 | 3 | 4 | 5 | 6 | 7 |
| **Positive Affect** | | | | | | | | |
| 1 | Interested |  |  |  |  |  |  |  |
| 2 | Excited |  |  |  |  |  |  |  |
| 3 | Strong |  |  |  |  |  |  |  |
| 4 | Enthusiastic |  |  |  |  |  |  |  |
| 5 | Proud |  |  |  |  |  |  |  |
| 6 | Alert |  |  |  |  |  |  |  |
| 7 | Inspired |  |  |  |  |  |  |  |
| 8 | Determined |  |  |  |  |  |  |  |
| 9 | Attentive |  |  |  |  |  |  |  |
| 10 | Active |  |  |  |  |  |  |  |
| **Negative Affect** | | | | | | | | |
| 11 | Distressed |  |  |  |  |  |  |  |
| 12 | Upset |  |  |  |  |  |  |  |
| 13 | Guilty |  |  |  |  |  |  |  |
| 14 | Scared |  |  |  |  |  |  |  |
| 15 | Hostile |  |  |  |  |  |  |  |
| 16 | Irritable |  |  |  |  |  |  |  |
| 17 | Ashamed |  |  |  |  |  |  |  |
| 18 | Nervous |  |  |  |  |  |  |  |
| 19 | Jittery |  |  |  |  |  |  |  |
| 20 | Afraid |  |  |  |  |  |  |  |
